# Supplementary material for: Redox-Dependent Modulation of Human Liver Progenitor Cell Line Fate
Source: Int J Mol Sci. 2023 Jan 18;24(3):1934. doi: 10.3390/ijms24031934 (PMC9916526; doi:10.3390/ijms24031934)
Supplement: Supplementary file 1 [file ijms-24-01934-s001.zip › ijms-2180236-supplementary.pdf]

Redox-dependent modulation of human liver progenitor cell line fate

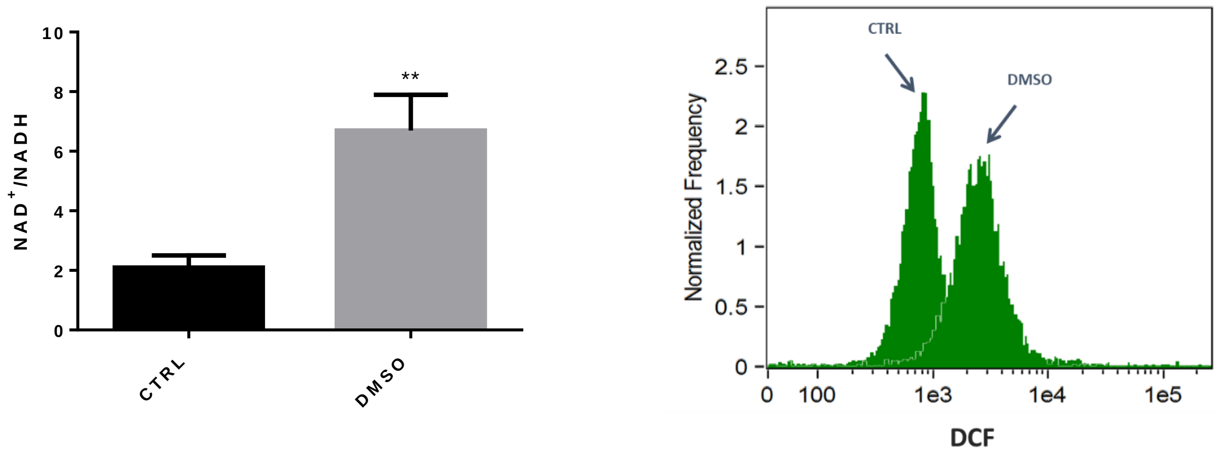

(a) (b)

Figure S1. (a) NAD+/NADH content in HepaRG cells in basal conditions (CTRL) or after 2 weeks exposure to dimethyl sulfoxide (DMSO). Data in the graph are represented as mean ± SEM of three independent experiments. Statistical differences were assessed by student's t-test. ** = $p < 0.01$ vs CTRL. **(b)** Flow cytometry histograms of HepaRG cells in basal conditions (CTRL) or after 2 weeks exposure to dimethyl sulfoxide (DMSO), after staining with dichlorofluorescein (DCF).

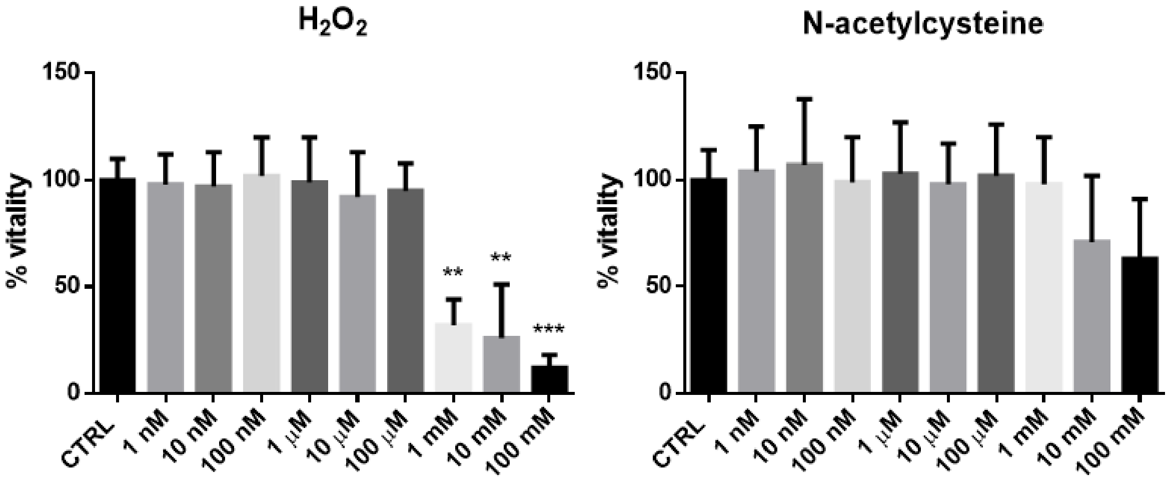

Figure S2. Cell viability of confluent HepaRG measured by 3-(4,5-dimethylthiazol-2-yl)-2,5diphenyltetrazolium bromide (MTT) reduction after 24 hours exposure to increasing concentrations of H_2O_2 or N-acetylcysteine. Data in the graph are represented as mean ± SEM of three independent experiments. Statistical differences were assessed by one-way ANOVA and Tukey-Kramer as post hoc test. ** = $p < 0.01$ vs CTRL; *** = $p < 0.001$ vs CTRL.

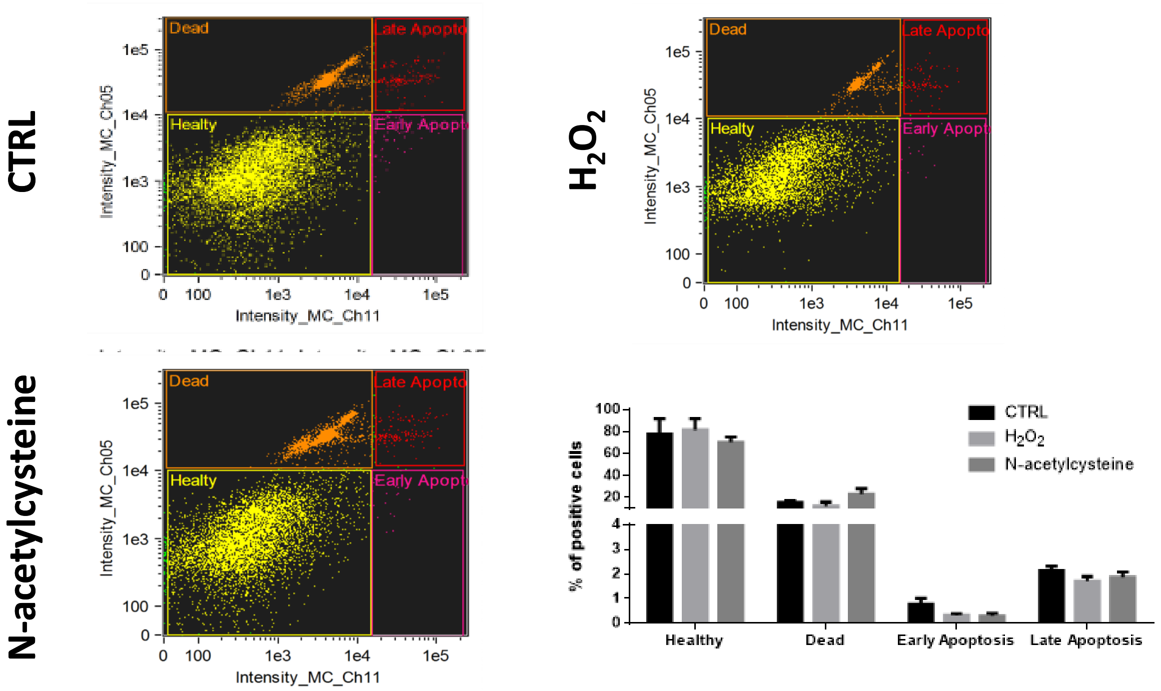

Figure S3. Flow cytometry scatter plots of apoptosis in confluent HepaRG exposed to H_2O_2 or N-acetylcysteine every 24 hours for 5 days, assessed after staining with annexin V (AV) and 7-Amino-Actinomycin D (7-AAD). Dead: AV negative/7-AAD positive cells (necrosis); Late Apoptosis: AV/7-AAD positive cells; Early Apoptosis: AV positive/7-AAD negative cells; Healthy: AV/7-AAD negative cells (viable). Data in the graphs are represented as mean ± SD of three independent experiments.

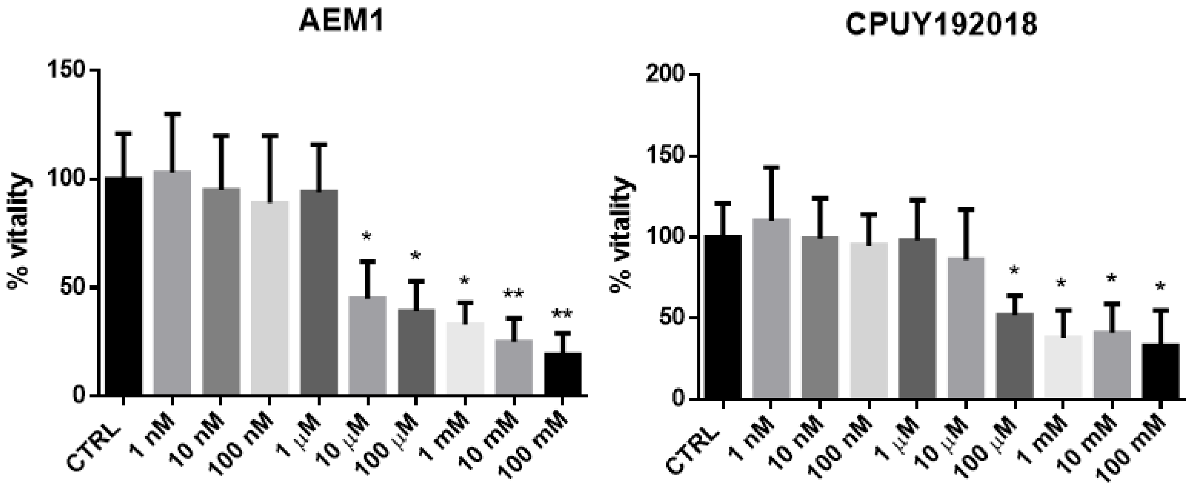

Figure S4. Cell viability of confluent HepaRG measured by 3-(4,5-dimethylthiazol-2-yl)-2,5diphenyltetrazolium bromide (MTT) reduction after 24 hours exposure to vehicle (CTRL), or increasing concentrations of ARE expression modulator 1 (AEM1) or CPUY192018. Data in the graph are represented as mean ± SEM of three independent experiments. Statistical differences were assessed by one-way ANOVA and Tukey-Kramer as post hoc test. * = $p < 0.05$ vs CTRL; ** = $p < 0.01$ vs CTRL.

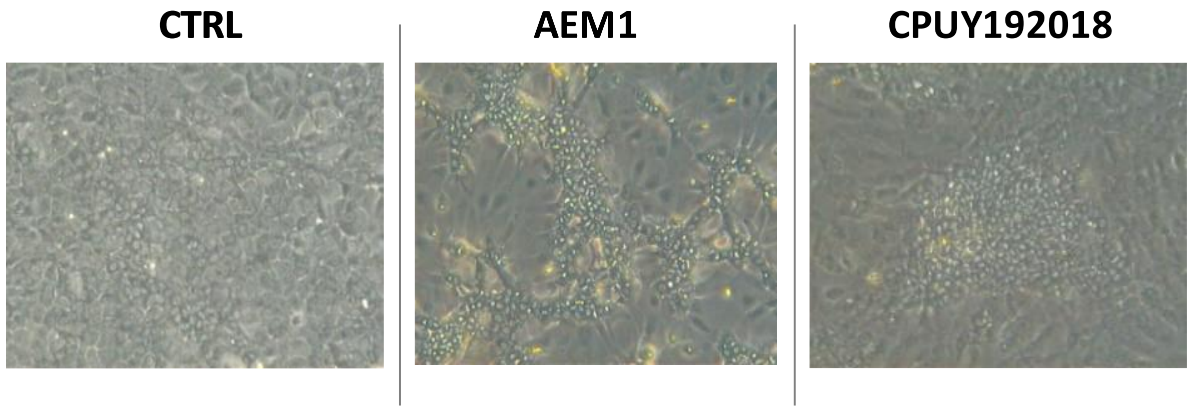

Figure S5. Representative microscopic images of confluent HepaRG cells exposed to vehicle (CTRL), 1 µM ARE expression modulator 1 (AEM1) or 10 µM CPUY192018 every 24 hours for 5 days.

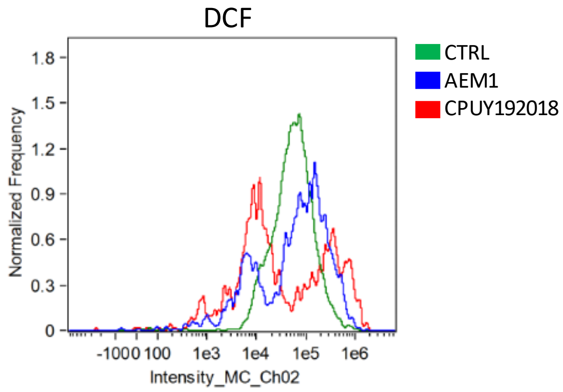

Figure S6. Flow cytometry histograms of confluent HepaRG cells exposed to vehicle (CTRL), 1 µM ARE expression modulator 1 (AEM1) or 10 µM CPUY192018 every 24 hours for 5 days, after staining with dichlorofluorescein (DCF).

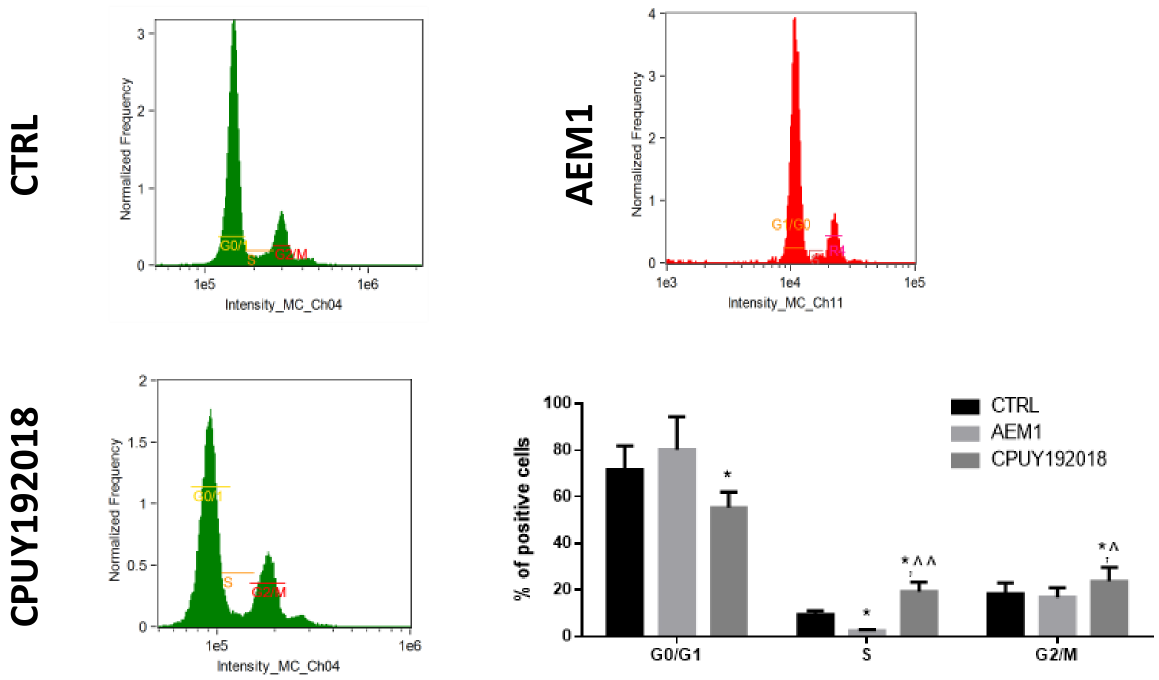

Figure S7. Cell cycle analysis in confluent HepaRG cells exposed to vehicle (CTRL), 1 μM ARE expression modulator or 10 μM CPUY 192018 every 24 hours for 5 days. Data in the graph are represented as mean ± SEM of three independent experiments. Statistical differences were assessed by one-way ANOVA and Tukey-Kramer as post hoc test. * = $p < 0.05$ vs CTRL; ^ = $p < 0.05$ vs AEM1; ^^ = $p < 0.01$ vs AEM1.

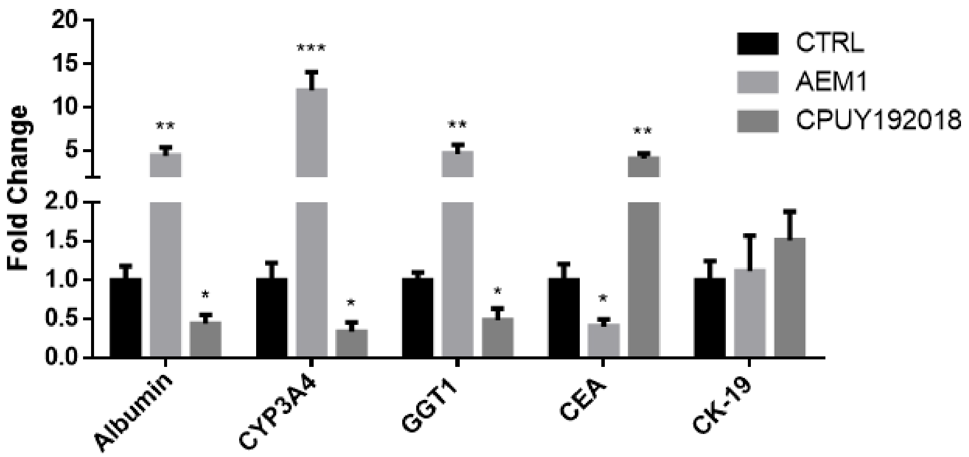

Figure S8. mRNA expression of genes associated with differentiation status in confluent HepaRG cells exposed to vehicle (CTRL), 1 μM ARE expression modulator 1 (AEM1) or 10 μM CPUY192018 every 24 hours for 5 days. Data in the graph are represented as mean ± SEM of three independent experiments. Statistical differences were assessed by one-way ANOVA and Tukey-Kramer as post hoc test. * = $p < 0.05$ vs CTRL; ** = $p < 0.01$ vs CTRL; *** = $p < 0.001$ vs CTRL.

Table S1. Sequences of forward (FOR) and reverse (REV) primers of the genes studied. CYP3A4: cytochrome P350 3A4; CEA, carcinoembryonic antigen; GGT1, gamma-glutamyl transpeptidase 1.

**ACTIN**	Human	FOR	5′-TGGACATCCGCAAAGACCTG-3′
		REV	5′-GCCGATCCACACGGAGTACTT-3′
**ALBUMIN**	Human	FOR	5′-CCTGTTGCCAAAGCTCGATG-3′
		REV	5′-GAAATCTCTGGCTCAGGCGA-3′
**CYP3A4**	Human	FOR	5′-CTTCATCCAATGGACTGCATAAAT-3′
		REV	5′-TCCCAAGTATAACACTCTACACAG-3′
**CEA**	Human	FOR	5′-GGTCTTCAACCCAATCAGTAAGAAC-3′
		REV	5′-ATGGCCCCAGGTGAGAGG-3′
**GGT1**	Human	FOR	5′-TTTGGTGTGCTGCTGGATGAC-3′
		REV	5′-ACCTGAGCTTCCCCACCTATG-3′
